# Correlation between Imaging Morphological Findings and Laboratory Biomarkers in Patients with Diabetic Macular Edema

**DOI:** 10.1155/2021/6426003

**Published:** 2021-08-13

**Authors:** Eleni Dimitriou, Theodoros N. Sergentanis, Vaia Lambadiari, George Theodossiadis, Panagiotis Theodossiadis, Irini Chatziralli

**Affiliations:** ^1^2nd Department of Ophthalmology, National and Kapodistrian University of Athens, Athens, Greece; ^2^Department of Clinical Therapeutics, Alexandra Hospital, Medical School, National and Kapodistrian University of Athens, Athens, Greece; ^3^2nd Department of Internal Medicine, Research Institute and Diabetes Center, National and Kapodistrian University of Athens, Athens, Greece

## Abstract

**Purpose:**

To investigate the potential association between peripheral blood biomarkers and morphological characteristics of retinal imaging in patients with diabetic macular edema (DME).

**Methods:**

Participants in this cross-sectional study were 36 consecutive patients (36 eyes) with treatment-naïve DME, who underwent spectral domain-optical coherence tomography (SD-OCT), fundus photography, and fundus fluorescein angiography (FFA). In addition, peripheral blood samples were taken to evaluate full blood count and biochemical parameters. Correlation between imaging characteristics and laboratory parameters was examined.

**Results:**

Eyes with central subfield thickness greater than 405 *μ*m presented significantly higher neutrophils/lymphocytes (*p* = 0.043) and higher lipoprotein (a) compared to eyes with CST < 405 *μ*m (*p* = 0.003). Presence of hyperreflective foci on SD-OCT was associated with significantly higher white blood cell count (*p* = 0.028). Ellipsoid zone disruption was associated with significantly lower hematocrit (*p* = 0.012), hemoglobin (*p* = 0.009), and red blood cell count (*p* = 0.026), as well as with higher lipoprotein (a) (*p* = 0.015). Macular ischemia on FFA was associated with significantly higher monocytes (*p* = 0.027) and monocytes/HDL (*p* = 0.019). No significant associations were found between laboratory parameters and subretinal fluid, intraretinal fluid, exudates, cysts, disorganization of inner retinal layers, epiretinal membrane, and external limiting membrane condition.

**Conclusion:**

Specific imaging morphological characteristics were found to be associated with laboratory parameters in patients with DME. These findings may shed light on the pathophysiology of DME and its correlation with the development of specific clinical signs.

## 1. Introduction

Diabetic macular edema (DME) is the most common cause of visual impairment in patients with diabetes mellitus (DM), characterized by exudation and accumulation of extracellular fluid in the macula [1, 2]. The overall prevalence of DME in patients with DM has been estimated to be about 7-14%, while it varies from 0% to 3% in patients with recent DM diagnosis and increases to 28% in patients with DM for more than 20 years [1–5].

In the pathogenesis of DME, chronic hyperglycemia promotes a cascade of biochemical pathways and consequent structural alterations in the retinal blood vessels' wall, including the loss of pericytes and the breakdown of the blood-retinal-barrier, leading to retinal vascular permeability [6, 7]. This breakdown is mainly driven by the production of inflammatory cytokines, with vascular endothelial growth factor (VEGF) to be the most prominent [6, 7]. Moreover, it has been shown that patients with DME have increased levels of proinflammatory mediators in aqueous humor compared to non-DME cases [8], while there is a controversy whether the pathophysiology of DME is mainly attributed to such systemic affection or to a local intraocular response. Of note, several studies have shown that elevated serum lipids, including cholesterol and low-density lipoprotein (LDL), demonstrated a significant association with retinal hard exudates and formation of DME [9–12], while elevated IL-6 has also been correlated with diffuse retinal thickness or severity of DME [13, 14].

Nowadays, there is a great development in retinal imaging, especially with the advent of spectral domain-optical coherence tomography (SD-OCT) and swept source-OCT (SS-OCT) [15, 16], as well as OCT angiography [17, 18]. Both OCT and OCTA are noninvasive techniques, enabling the identification of specific morphological characteristics of DME, while OCTA allows the quantification of the foveal avascular zone (FAZ) area besides vessel density [18].

Given the current understanding of DME pathogenesis, although much reported information exists on intraocular biomarkers in patients with DME, literature is scarce regarding systemic biomarkers and their correlation with morphological characteristics in retinal imaging. Ghosh et al. examined the relationship between different OCT patterns of DME and systemic risk factors in patients with DME and did not identify any modifiable systemic factor for any of the OCT patterns in DME [19].

Based on the above, the purpose of the present study was to investigate the potential association between peripheral blood biomarkers and morphological characteristics of retinal imaging in patients with DME.

## 2. Methods

Participants in this observational, cross-sectional study were 36 consecutive patients with DM type 2 and treatment naïve DME, who were diagnosed and treated at the 2nd Department of Ophthalmology, National and Kapodistrian University of Athens, Athens, Greece, between 1st September 2019 and 31st March 2020. The study protocol adhered to the tenets of the Declaration of Helsinki and was approved by the Institutional Review Board. Written informed consent was obtained from all participants.

All patients had nonproliferative diabetic retinopathy (NPDR) and treatment naïve DME, confirmed on SD-OCT, revealing central subfield thickness (CST) ≥ 320 *μ*m. One eye of each patient was included. In cases of bilateral DME, the right eye was chosen, so as to avoid selection bias. Patients with other vitreoretinal diseases, uveitis, media opacities, previous vitreoretinal surgery, previous laser photocoagulation, or ocular surgery in the previous 6 months were excluded.

Demographic data of patients (age, gender) were recorded, along with the duration of DM. All participants underwent a complete ophthalmologic examination, including best-corrected visual acuity (BCVA) measurement by means of Snellen's charts (converted to logMAR scale), slit-lamp examination, dilated fundoscopy, color fundus photography using Topcon TRC-50DX (Topcon Corporation), SD-OCT, and fundus fluorescein angiography (FFA) using Spectralis (Spectralis HRA+OCT, Heidelberg Engineering, Heidelberg, Germany). SD-OCT was obtained using a standard acquisition protocol; six radial scans 3 mm long were performed at equally spaced angular orientations centered on the foveola. The OCT volume scan was performed on a 20 × 20 degree cube, consisted of 49 horizontal B-scans with 20 averaged frames per B-scan centered over the fovea. The following SD-OCT variables were recorded at baseline: CST (*μ*m), presence of intraretinal fluid (IRF), subretinal fluid (SRF), cysts, hyperreflective foci (HF), and disorganization of the inner retinal layers (DRIL) and epiretinal membrane (ERM). Ellipsoid zone (EZ) and external limiting membrane (ELM) condition were also assessed. In addition, the presence of exudates on color fundus photography was recorded. The severity of DR was based on color fundus photography and on FFA and was graded according to the international diabetic retinopathy disease severity scale [20]. Moreover, macular ischemia was evaluated on FFA and defined as disruption and enlargement of foveal avascular zone (FAZ). Two investigators (IC, ED) independently evaluated qualitatively the SD-OCT images, the fundus photographs, and the FFA images. The interobserver agreement ranged from very good to perfect for all SD-OCT parameters (*k* = 0.999 for IRF; *k* = 0.999 for SRF; *k* = 0.872 for HF; *k* = 0.851 for DRIL; *k* = 0.999 for ERM; *k* = 0.902 for EZ condition; and *k* = 0.883 for ELM condition), as well as for DR severity assessment on fundus photographs (*k* = 0.901) and ischemia evaluation on FFA (*k* = 0.935).

At the same day and following an eight hour overnight fast, all patients underwent a forearm venous puncture for peripheral blood extraction and serum was separated. Full blood count was measured on a Sysmex XE-2100 analyzer (Sysmex Corp. Kobe, Japan), while all biochemical analyses were performed on a Roche Cobas 8000 (Roche, Chicago, IL, USA) in the laboratory of Attikon University Hospital. Specifically, we analyzed the following parameters: glucose, glycated Hb (HbA1c), urea, creatinine, cholesterol, HDL, LDL, triglycerides, apolipoprotein A, apolipoprotein B, lipoprotein (a), homocysteine, vitamin D, and IL-6.

### 2.1. Statistical Analysis

For the description of patients' characteristics, descriptive statistics were calculated; mean ± standard deviation (SD) was used for continuous variables, while relative frequencies and percentages for categorical variables were reported. All variables were tested for normal distribution with the Shapiro–Wilk test. The associations between laboratory and imaging variables were evaluated with the Mann–Whitney–Wilcoxon test (MWW), as appropriate. In addition, Pearson's chi-squared test (P) or Fisher's exact test (F) were also appropriately implemented. According to the *a priori* power calculation, a sample size of 36 eyes was adequate to achieve 80% power for the detection of an effect size larger or equal to 1.05 (in simple terms, a difference of 1.05∗SD between the compared subgroups), assuming an application of the two-tailed Mann–Whitney–Wilcoxon test for equally sized subgroups at the 5% level of significance. The sample size calculation was performed with G∗Power 3.1.9.2 software (University of Dusseldorf, Germany). Statistical analysis was performed using STATA/SE 13 statistical software (Stata Corporation, College Station, TX, USA). A *p* value < 0.05 was considered statistically significant.

## 3. Results

The demographic and clinical characteristics of the study sample are shown in Table 1. The mean age of patients was 64.2 ± 8.5 years. 58.3% of patients were male and 41.7% female. The mean duration of DM was 11.7 ± 4.7 years. The mean BCVA was 0.51 ± 0.29 logMAR, while the mean CST was 439.2 ± 79.1 *μ*m.

Regarding the potential association between imaging characteristics and laboratory variables, no significant correlations were found for IRF, SRF, exudates, cysts, DRIL, ERM, and ELM condition.

Table 2 shows the comparison of laboratory parameters between eyes with HF (*n* = 15) and without HF (*n* = 21). Eyes with HF presented significantly higher white blood cell (WBC) count compared to those without HF (*p* = 0.028, MWW).

Table 3 shows the comparison of laboratory parameters between eyes with intact EZ (*n* = 23) and those with disrupted EZ (*n* = 13). Eyes with disrupted EZ presented significantly lower hematocrit (*p* = 0.012, MWW) and hemoglobin (Hb) (*p* = 0.009, MWW), as well as lower red blood cell (RBC) count (*p* = 0.026, MWW), while they had significantly higher lipoprotein (a) compared to eyes with intact EZ (*p* = 0.015, MWW).

Table 4 shows the comparison of laboratory parameters between eyes with macular ischemia (*n* = 9) and those without macular ischemia (*n* = 27). Eyes with macular ischemia had significantly higher monocytes (*p* = 0.027, MWW) and monocytes/HDL (*p* = 0.019, MWW) compared to eyes without macular ischemia.

Table 5 shows the comparison of laboratory parameters between eyes with CST above or equal to median (405 *μ*m) and those with CST below median. Eyes with CST ≥ 405 *μ*m presented higher neutrophils/lymphocytes (*p* = 0.043, MWW) and higher lipoprotein (a) compared to eyes with CST < 405 *μ*m (*p* = 0.003, MWW).

**Figure 1:**
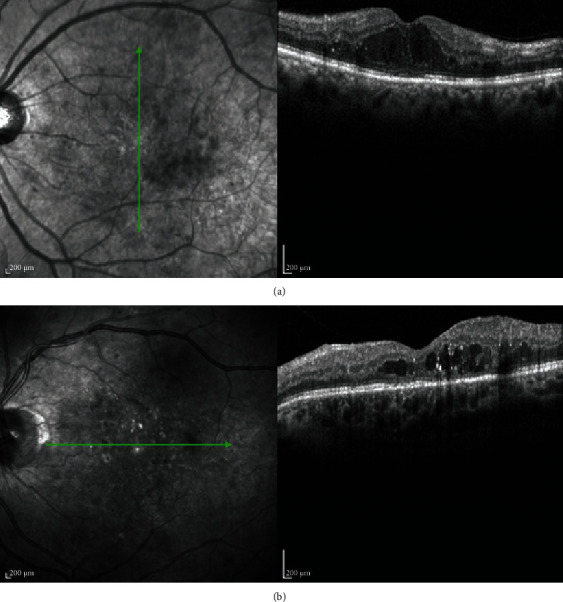
(A) Optical coherence tomography of a female patient with diabetic macular edema and elevated lipoprotein (a), as well as decreased hematocrit and red blood count, which was associated with the disruption of the ellipsoid zone and an increase in central subfield thickness. (B) Optical coherence tomography of a male patient with diabetic macular edema and elevated white blood cells, which were associated with the presence of hyperreflective foci.

**Figure 2:**
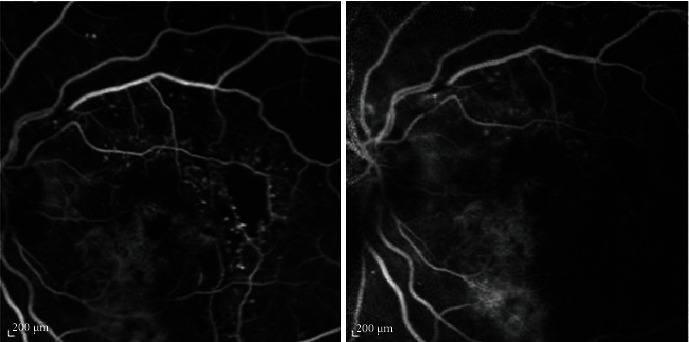
Fluorescein angiography of a male patient with diabetic macular edema, showing macular ischemia, which was associated with higher monocytes and higher monocytes/HDL.

Figures 1 and 2 show correlation between laboratory and morphological findings in patients with DME.

## 4. Discussion

The principal message of the study is that most of the imaging morphological characteristics studied herein were not correlated with laboratory parameters in patients with DME, suggesting that DME may be mainly attributed to a local response more than a systemic effect. However, CST and specific OCT biomarkers, i.e., HF and EZ conditions, as well as macular ischemia on FFA, were found to be associated with laboratory findings in patients with DME, shedding light on the pathophysiology of DME and its correlation with the development of these specific clinical characteristics.

Firstly, lipoprotein (a) was found to be associated with high CST and with EZ disruption. Previous studies have shown that high lipoprotein (a) concentration was independently associated with the presence and severity of DR in patients with type 2 DM, regardless of glycemic control [21–25]. Specifically, lipoprotein (a) can affect vascular tone and perfusion, oxidize lipids, and enhance oxidative stress via the generation of reactive oxygen species and inflammatory actions on the vascular wall [26, 27]. Moreover, lipoprotein (a) has been associated with endothelial dysfunction, suggesting that it could be an independent risk factor for diabetic microvascular complications [28, 29]. Although the exact mechanism behind the potential causal relationship between lipoprotein (a) and DME, and especially EZ disruption, remains unclear, it has been hypothesized that elevated lipoprotein (a) concentrations may play a causative role in DME by damaging the microcirculation [30]. In addition, lipoprotein (a) has been involved in the activation of acute inflammation and may be related to more severe DR, including DME severity with higher CST and EZ disruption [31]. Ellipsoid zone disruption has also been associated with decreased RBC count, decreased Hb, and consequently, decreased hematocrit. In patients with DM, RBC have been shown to present variations in size and diameter [32]. Chronic hyperglycemia can lead to nonenzymatic glycation of RBC membrane proteins that would accelerate RBC aging due to negative surface electrical charge [32, 33]. The RBC count has been found to be reduced in patients with microvascular complications, especially in those with longer DM duration [32–34]. Since Hb levels are directly correlated with RBC count, reduced RBC would reflect on Hb concentration and on hematocrit. In our case with DME, hyperglycemia induces the rearrangement of proteins in the plasma membrane, while cytoskeleton proteins also appear to be heavily glycosylated, affecting membrane stability, as it has been mentioned in RBC membrane proteins [32]. Therefore, reduced RBC could represent more severe DR and DME, which may be reflected on EZ disruption, as a result of cytoskeleton weakening.

An interesting finding of our study was the association between HF and WBC. Many theories have attempted to explain the pathophysiology of HF, but their precise nature remains elusive. Framme et al. suggested that HF can be leucocytes or RPE cells, indicating retinal inflammation [35]. Coscas et al. supported this concept and postulated that HF are microglia cells activated by inflammation [36]. White blood cells and their subtypes are the biomarkers of inflammatory response because their activation leads to the synthesis of inflammatory cytokines, as it has been previously identified in patients with DR [37, 38]. Therefore, our finding that HF presence was associated with increased WBC is consistent with the hypothesis that an inflammatory component is implemented in HF pathogenesis [39–41].

In recent studies, neutrophil (an indicator of inflammation) to lymphocyte (an indicator of physiologic stress) ratio was evaluated in inflammatory diseases, such as coronary artery disease, noncardiac diseases, retinal vein occlusion, age-related macular degeneration and DR [42–47]. Of note, neutrophil-to-lymphocyte ratio is more powerful for prediction of inflammatory diseases than subtypes of WBC alone because it combines the predictive values of two parameters of WBC [44]. In DME, the chronic low-grade inflammation may lead to inflammatory cytokine release, which is commonly responsible for increased vascular permeability [7]. As a result, neutrophil-to-lymphocyte ratio may be defined as an indication of subclinical inflammation, especially in more severe DME, as it was found in our study, showing a significant association between increased neutrophil-to-lymphocyte ratio and higher CST.

Moreover, monocyte-to-HDL ratio has been investigated as a new inflammation biomarker and is considered superior to subtypes of WBC, especially in patients with cardiovascular and cerebrovascular diseases, as well as in patients with branch retinal vein occlusion [48–50]. Monocytes are indicators of inflammation, since they are responsible for inflammatory cytokine secretion, while HDL has antioxidant and anti-inflammatory effects [51, 52]. In our study, patients with macular ischemia were found to have increased monocytes, as well as monocyte-to-HDL ratio, which is consistent with other studies in patients with myocardial infarction and limb ischemia, suggesting monocyte-to-HDL ratio as a biomarker of ischemic conditions [53, 54].

A potential limitation of this study pertains to the relatively small sample size, although our *a priori* statistical power calculation showed that it was adequate to achieve 80% power for the detection of an effect size larger or equal to 1.05; further larger studies seem necessary to validate our results.

In conclusion, this study investigated the potential correlation between imaging morphological findings and laboratory biomarkers in patients with DME. Our results showed that specific inflammatory biomarkers, such as WBC, monocytes, monocyte-to-HDL, and neutrophil-to-lymphocyte ratios, were associated with more severe disease activity with higher CST and presence of HF and macular ischemia, while EZ disruption was found to be associated with increased lipoprotein (a) and decreased RBC, both of which were involved in microcirculation alterations in patients with DME. These findings may scrutinize the pathophysiology of DME and the pathogenesis of specific clinical signs. However, it should be noted that most of imaging biomarkers studied herein were not correlated with laboratory parameters in patients with DME, suggesting that DME may be mainly attributed to a local response more than a systemic effect. Further studies with a large sample size are needed to justify our results.

## Figures and Tables

**Table 1:** Demographic and clinical characteristics of the study sample (*n* = 36 patients with diabetic macular edema).

Age (mean ± SD, years)	64.2 ± 8.5
Gender (*n*, %)	
Male	21 (58.3%)
Female	15 (41.7%)
Duration of diabetes mellitus (mean ± SD, years)	11.7 ± 4.7
HbA1c (mean ± SD, %)	8.4 ± 1.9
Stage of diabetic retinopathy (*n*, %)	
Mild	10 (27.8%)
Moderate	17 (47.2%)
Severe	9 (25.0%)
Best-corrected visual acuity (mean ± SD, logMAR)	0.51 ± 0.29
Imaging characteristics	
Central subfield thickness (mean ± SD, *μ*m)	439.2 ± 79.1
Intraretinal fluid (*n*, %)	36 (100%)
Subretinal fluid (*n*, %)	9 (25%)
Cysts (*n*, %)	2 (5.6%)
Hyperreflective foci (*n*, %)	15 (41.7%)
Exudates (*n*, %)	13 (36.1%)
Disorganization of inner retinal layer (*n*, %)	6 (16.7%)
Epiretinal membrane (*n*, %)	2 (5.6%)
Ellipsoid zone condition (*n*, %)	
Intact	23 (63.9%)
Disrupted	13 (36.1%)
External limiting membrane condition (*n*, %)	
Intact	26 (72.2%)
Disrupted	10 (27.8%)
Macular ischemia (*n*, %)	9 (25%)

**Table 2:** Association between laboratory variables and hyperreflective foci on optical coherence tomography. Laboratory variables are summarized as median (IQR: interquartile range).

	HF present (*n* = 15)	HF absent (*n* = 21)	*p* value (MWW)
Red blood cells (10^6^/*μ*l)	4.65 (0.61)	4.73 (0.91)	0.824
White blood cells (10^3^/*μ*l)	8.54 (2.10)	7.29 (2.26)	**0.028**
Neutrophils (10^3^/*μ*l)	5.24 (2.08)	4.37 (1.41)	0.124
Lymphocytes (10^3^/*μ*l)	2.30 (1.01)	1.91 (0.61)	0.096
Monocytes (10^3^/*μ*l)	0.63 (0.20)	0.51 (0.27)	0.260
Platelets (10^3^/*μ*l)	248 (37)	219 (78)	0.073
Hematocrit (%)	40.6 (4.1)	40.7 (3.5)	0.547
Hemoglobin (g/dl)	13.9 (1.5)	13.4 (1.5)	0.419
Monocytes/lymphocytes	0.27 (0.14)	0.29 (0.19)	0.962
Neutrophils/lymphocytes	2.15 (0.92)	2.25 (1.36)	0.937
Monocytes/HDL	0.013 (0.006)	0.011 (0.007)	0.516
Glucose (mg/dl)	198 (87)	161 (81)	0.084
HbA1c (%)	8.8 (2.5)	7.8 (1.8)	0.281
Urea (mg/dl)	37.8 (10.8)	37.5 (23.6)	0.635
Creatinine (mg/dl)	0.8 (0.2)	0.9 (0.7)	0.168
Cholesterol (mg/dl)	172 (61)	147 (56)	0.384
LDL (mg/dl)	94 (56)	80 (44)	0.310
HDL (mg/dl)	45 (14)	47 (17)	0.975
Triglycerides (mg/dl)	170 (168)	122 (89)	0.059
IL-6 (pg/ml)	3.5 (3.7)	3.8 (2.2)	0.680
Apolipoprotein A (mg/dl)	145 (35)	141 (37)	0.334
Apolipoprotein B (mg/dl)	89 (44)	77 (34)	0.228
Lipoprotein (a) (nmol/l)	28.7 (38.6)	15.4 (107.3)	0.975
Homocysteine (*μ*mol/l)	14.7 (7.4)	17.8 (11.2)	0.141
Vitamin D (ng/ml)	19.8 (12.7)	21.6 (18.1)	0.506

HF: hyperreflective foci; MWW: Mann–Whitney–Wilcoxon's test.

**Table 3:** Association between laboratory variables and ellipsoid zone condition on optical coherence tomography. Laboratory variables are summarized as median (IQR: interquartile range).

	EZ intact (*n* = 23)	EZ disrupted (*n* = 13)	*p* value (MWW)
Red blood cells (10^6^/*μ*l)	4.75 (0.71)	4.38 (0.53)	**0.026**
White blood cells (10^3^/*μ*l)	7.81 (3.01)	8.09 (2.28)	0.417
Neutrophils (10^3^/*μ*l)	4.64 (2.03)	5.29 (3.02)	0.174
Lymphocytes (10^3^/*μ*l)	2.04 (0.62)	2.18 (1.00)	0.846
Monocytes (10^3^/*μ*l)	0.54 (0.22)	0.57 (0.33)	0.818
Platelets (10^3^/*μ*l)	246 (71)	241 (31)	0.737
Hematocrit (%)	41.2 (4.4)	38.7 (3.7)	**0.012**
Hemoglobin (g/dl)	14.0 (1.2)	12.9 (1.3)	**0.009**
Monocytes/lymphocytes	0.27 (0.14)	0.28 (0.22)	0.659
Neutrophils/lymphocytes	2.25 (1.25)	2.44 (1.53)	0.548
Monocytes/HDL	0.013 (0.006)	0.013 (0.007)	>0.999
Glucose (mg/dl)	177 (87)	113 (105)	0.133
HbA1c (%)	7.6 (2.3)	9.0 (1.5)	0.331
Urea (mg/dl)	36.2 (17.4)	42.3 (18.3)	0.274
Creatinine (mg/dl)	0.8 (0.2)	1.0 (0.7)	0.143
Cholesterol (mg/dl)	153 (64)	166 (39)	0.584
LDL (mg/dl)	86 (55)	87 (43)	0.902
HDL (mg/dl)	45 (19)	50 (11)	0.584
Triglycerides (mg/dl)	131 (149)	138 (120)	0.596
IL-6 (pg/ml)	3.4 (2.1)	4.5 (8.0)	0.377
Apolipoprotein A (mg/dl)	142 (36)	143 (13)	0.750
Apolipoprotein B (mg/dl)	82 (42)	84 (25)	0.724
Lipoprotein (a) (nmol/l)	14.7 (34.8)	89.5 (188.4)	**0.015**
Homocysteine (*μ*mol/l)	15.2 (8.0)	18.9 (8.7)	0.197
Vitamin D (ng/ml)	23 (18.1)	25.4 (9.8)	0.090

EZ: ellipsoid zone; MWW: Mann–Whitney–Wilcoxon's test.

**Table 4:** Association between laboratory variables and macular ischemia on fluorescein angiography. Laboratory variables are summarized as median (IQR: interquartile range).

	Macular ischemia presence (*n* = 9)	Macular ischemia absence (*n* = 27)	*p* (MWW)
Red blood cells (10^6^/*μ*l)	4.65 (0.73)	4.70 (0.82)	0.932
White blood cells (10^3^/*μ*l)	9.0 (2.49)	7.57 (1.96)	0.096
Neutrophils (10^3^/*μ*l)	5.24 (1.96)	4.57 (2.03)	0.074
Lymphocytes (10^3^/*μ*l)	2.33 (1.87)	2.03 (0.46)	0.718
Monocytes (10^3^/*μ*l)	0.64 (0.38)	0.51 (0.19)	**0.027**
Platelets (10^3^/*μ*l)	243 (86)	244 (72)	0.430
Hematocrit (%)	40.5 (6.4)	40.7 (5.3)	0.503
Hemoglobin (g/dl)	13.2 (1.4)	13.9 (1.4)	0.345
Monocytes/lymphocytes	0.29 (0.21)	0.27 (0.14)	0.159
Neutrophils/lymphocytes	2.15 (2.95)	2.25 (0.99)	0.718
Monocytes/HDL	0.016 (0.010)	0.011 (0.004)	**0.019**
Glucose (mg/dl)	198 (219)	167 (62)	0.606
HbA1c (%)	9.4 (3.0)	7.4 (1.8)	0.074
Urea (mg/dl)	40 (16.8)	35.8 (20)	0.154
Creatinine (mg/dl)	0.8 (0.5)	0.8 (0.2)	0.889
Cholesterol (mg/dl)	172 (80)	147 (55)	0.216
LDL (mg/dl)	98 (68)	80 (43)	0.287
HDL (mg/dl)	43 (16)	48 (19)	0.390
Triglycerides (mg/dl)	211 (190)	128 (70)	0.198
IL-6 (pg/ml)	4.2 (6.2)	3.0 (2.5)	0.149
Apolipoprotein A (mg/dl)	141 (33)	143 (34)	0.606
Apolipoprotein B (mg/dl)	93 (63)	81 (33)	0.363
Lipoprotein (a) (nmol/l)	34.7 (92.6)	15.4 (59.6)	0.363
Homocysteine (*μ*mol/l)	17.8 (6.8)	15.7 (8)	0.731
Vitamin D (ng/ml)	17.9 (13)	21.6 (12.3)	0.668

MWW: Mann–Whitney–Wilcoxon's test.

**Table 5:** Association between laboratory variables and central subfield thickness on optical coherence tomography. Laboratory variables are summarized as median (IQR: interquartile range).

	Central subfield thickness ≥ 405 *μ*m (*n* = 18)	Central subfield thickness < 405 *μ*m (*n* = 18)	*p* (MWW)
Red blood cells (10^6^/*μ*l)	4.6 (0.48)	4.79 (0.76)	0.097
White blood cells (10^3^/*μ*l)	7.7 (3.31)	7.87 (1.95)	0.728
Neutrophils (10^3^/*μ*l)	5.0 (3.0)	4.64 (1.74)	0.174
Lymphocytes (10^3^/*μ*l)	1.97 (0.76)	2.08 (1.02)	0.282
Monocytes (10^3^/*μ*l)	0.57 (0.29)	0.54 (0.20)	0.824
Platelets (10^3^/*μ*l)	241 (82)	251 (68)	0.359
Hematocrit (%)	40.5 (4.7)	40.9 (4.1)	0.728
Hemoglobin (g/dl)	13.3 (1.5)	13.9 (1.0)	0.516
Monocytes/lymphocytes	0.28 (0.20)	0.27 (0.17)	0.268
Neutrophils/lymphocytes	2.74 (1.34)	1.95 (0.72)	**0.043**
Monocytes/HDL	0.012 (0.007)	0.013 (0.006)	0.950
Glucose (mg/dl)	159 (92)	180 (97)	0.179
HbA1c (%)	8.2 (2.3)	7.6 (3.4)	0.924
Urea (mg/dl)	37 (19)	38.9 (17.1)	0.812
Creatinine (mg/dl)	0.9 (0.5)	0.8 (0.2)	0.608
Cholesterol (mg/dl)	163 (47)	153 (64)	0.658
LDL (mg/dl)	95 (43)	84 (55)	0.716
HDL (mg/dl)	48 (12)	45 (20)	0.924
Triglycerides (mg/dl)	137 (90)	122 (207)	0.693
IL-6 (pg/ml)	3.4 (3.6)	3.8 (1.9)	0.764
Apolipoprotein A (mg/dl)	142 (32)	144 (43)	0.937
Apolipoprotein B (mg/dl)	88 (25)	75 (56)	0.359
Lipoprotein (a) (nmol/l)	60.0 (162.1)	10.1 (17.4)	**0.003**
Homocysteine (*μ*mol/l)	18.3 (10.9)	15.2 (6.9)	0.624
Vitamin D (ng/ml)	21.7 (13.0)	21.3 (18.7)	0.776

MWW: Mann–Whitney–Wilcoxon's test.

## Data Availability

Data are available upon request from authors.

## References

[1] Klein R., Klein B. E., Moss S. E., Davis M. D., DeMets D. L. (1984). The Wisconsin Epidemiologic Study of Diabetic Retinopathy: IV. Diabetic Macular Edema. *Ophthalmology*.

[2] Klein R., Klein B. E., Moss S. E. (1984). Visual impairment in diabetes. *Ophthalmology*.

[3] Yau J. W., Rogers S. L., Kawasaki R. (2012). Global prevalence and major risk factors of diabetic retinopathy. *Diabetes Care*.

[4] Klein R., Klein B. E., Moss S. E., Cruickshanks K. J. (1995). The Wisconsin Epidemiologic Study of Diabetic Retinopathy XV: The Long-term Incidence of Macular Edema. *Ophthalmology*.

[5] Zhang X., Saaddine J. B., Chou C. F. (2010). Prevalence of diabetic retinopathy in the United States, 2005-2008. *JAMA*.

[6] Das A., McGuire P. G., Rangasamy S. (2015). Diabetic macular edema: pathophysiology and novel therapeutic targets. *Ophthalmology*.

[7] Romero-Aroca P., Baget-Bernaldiz M., Pareja-Rios A., Lopez-Galvez M., Navarro-Gil R., Verges R. (2016). Diabetic macular edema pathophysiology: vasogenic versus inflammatory. *Journal Diabetes Research*.

[8] Dong N., Xu B., Chu L., Tang X. (2015). Study of 27 aqueous humor cytokines in type 2 diabetic patients with or without macular edema. *PLoS One*.

[9] Sabaner M. C., Akdogan M., Doğan M. (2020). Inflammatory cytokines, oxidative and antioxidative stress levels in patients with diabetic macular edema and hyperreflective spots. *European Journal of Ophthalmology*.

[10] Crosby-Nwaobi R., Chatziralli I., Sergentanis T., Dew T., Forbes A., Sivaprasad S. (2015). Cross talk between lipid metabolism and inflammatory markers in patients with diabetic retinopathy. *Journal Diabetes Research*.

[11] Sasaki M., Kawasaki R., Noonan J. E., Wong T. Y., Lamoureux E., Wang J. J. (2013). Quantitative measurement of hard exudates in patients with diabetes and their associations with serum lipid levels. *Investigative Ophthalmology & Visual Science*.

[12] Benarous R., Sasongko M. B., Qureshi S. (2011). Differential association of serum lipids with diabetic retinopathy and diabetic macular edema. *Investigative Ophthalmology & Visual Science*.

[13] Figueras-Roca M., Molins B., Sala-Puigdollers A. (2017). Peripheral blood metabolic and inflammatory factors as biomarkers to ocular findings in diabetic macular edema. *PLoS One*.

[14] Figueras-Roca M., Matas J., Llorens V. (2021). Systemic contribution of inflammatory mediators to the severity of diabetic and uveitic macular edema. *Graefe's Archive for Clinical and Experimental Ophthalmology*.

[15] Phadikar P., Saxena S., Ruia S., Lai T. Y., Meyer C. H., Eliott D. (2017). The potential of spectral domain optical coherence tomography imaging based retinal biomarkers. *International Journal of Retina and Vitreous*.

[16] Vira J., Marchese A., Singh R. B., Agarwal A. (2020). Swept-source optical coherence tomography imaging of the retinochoroid and beyond. *Expert Review of Medical Devices*.

[17] Cicinelli M. V., Cavalleri M., Brambati M., Lattanzio R., Bandello F. (2019). New imaging systems in diabetic retinopathy. *Acta Diabetologica*.

[18] Tey K. Y., Teo K., Tan A. C. S. (2019). Optical coherence tomography angiography in diabetic retinopathy: a review of current applications. *Eye and Vision*.

[19] Ghosh S., Bansal P., Shejao H., Hegde R., Roy D., Biswas S. (2015). Correlation of morphological pattern of optical coherence tomography in diabetic macular edema with systemic risk factors in middle aged males. *International Ophthalmology*.

[20] Wilkinson C. P., Ferris FL 3rd, Klein R. E. (2003). Proposed international clinical diabetic retinopathy and diabetic macular edema disease severity scales. *Ophthalmology*.

[21] Chopra Y. J. S., Lim T. S., Cha S. A. (2016). Lipoprotein(a) predicts the development of diabetic retinopathy in people with type 2 diabetes mellitus. *Journal of Clinical Lipidology*.

[22] Funatsu H., Shimizu E., Noma H., Mimura T., Hori S. (2009). Association between serum lipoprotein (a) level and progression of non-proliferative diabetic retinopathy in type 2 diabetes. *Acta Ophthalmologica*.

[23] Chopra R., Saramma J. G., Mary J., Rebecca A. (2007). Lipoprotein(a) as a risk factor for diabetic retinopathy in patients with type 2 diabetes mellitus. *Indian Journal of Ophthalmology*.

[24] Guerci B., Meyer L., Sommer S. (1999). Severity of diabetic retinopathy is linked to lipoprotein (a) in type 1 diabetic patients. *Diabetes & Metabolism*.

[25] Tu W. J., Liu H., Liu Q., Cao J. L., Guo M. (2017). Association between serum lipoprotein(a) and diabetic retinopathy in Han Chinese patients with type 2 diabetes. *The Journal of Clinical Endocrinology and Metabolism*.

[26] Sotiriou S. N., Orlova V. V., Al-Fakhri N. (2006). Lipoprotein(a) in atherosclerotic plaques recruits inflammatory cells through interaction with Mac-1 integrin. *The FASEB Journal*.

[27] Tsimikas S., Hall J. L. (2012). Lipoprotein(a) as a potential causal genetic risk factor of cardiovascular disease: a rationale for increased efforts to understand its pathophysiology and develop targeted therapies. *Journal of the American College of Cardiology*.

[28] Wu H. D., Berglund L., Dimayuga C. (2004). High lipoprotein(a) levels and small apolipoprotein(a) sizes are associated with endothelial dysfunction in a multiethnic cohort. *Journal of the American College of Cardiology*.

[29] Jenkins A. J., Lyons T. J., Zheng D. (2003). Lipoproteins in the DCCT/EDIC cohort: associations with diabetic nephropathy. *Kidney International*.

[30] Woodman R. J., Watts G. F., Playford D. A., Best J. D., Chan D. C. (2005). Oxidized LDL and small LDL particle size are independently predictive of a selective defect in microcirculatory endothelial function in type 2 diabetes. *Diabetes, Obesity & Metabolism*.

[31] Muni R. H., Kohly R. P., Lee E. Q., Manson J. E., Semba R. D., Schaumberg D. A. (2013). Prospective study of inflammatory biomarkers and risk of diabetic retinopathy in the diabetes control and complications trial. *JAMA Ophthalmology*.

[32] Alamri B. N., Bahabri A., Aldereihim A. A. (2019). Hyperglycemia effect on red blood cells indices. *European Review for Medical and Pharmacological Sciences*.

[33] Wang Y., Yang P., Yan Z. (2021). The relationship between erythrocytes and diabetes mellitus. *Journal Diabetes Research*.

[34] Buys A. V., Van Rooy M. J., Soma P., Van Papendorp D., Lipinski B., Pretorius E. (2013). Changes in red blood cell membrane structure in type 2 diabetes: a scanning electron and atomic force microscopy study. *Cardiovascular Diabetology*.

[35] Framme C., Wolf S., Wolf-Schnurrbusch U. (2010). Small dense particles in the retina observable by spectral-domain optical coherence tomography in age-related macular degeneration. *Investigative Ophthalmology & Visual Science*.

[36] Coscas G., Coscas F., Vismara S., Souied E., Soubrane G. (2008). Tomographie par coherence optique de type Spectral Domain dans la degenerescence maculaire liee a l’age:. *Journal Français d'Ophtalmologie*.

[37] Fest J., Ruiter R., Ikram M. A., Voortman T., van Eijck C. H. J., Stricker B. H. (2018). Reference values for white blood-cell-based inflammatory markers in the Rotterdam Study: a population-based prospective cohort study. *Scientific Reports*.

[38] Rangasamy S., McGuire P. G., Franco Nitta C., Monickaraj F., Oruganti S. R., Das A. (2014). Chemokine mediated monocyte trafficking into the retina: role of inflammation in alteration of the blood-retinal barrier in diabetic retinopathy. *PLoS One*.

[39] Vujosevic S., Midena E. (2013). Retinal layers changes in human preclinical and early clinical diabetic retinopathy support early retinal neuronal and Müller cells alterations. *Journal Diabetes Research*.

[40] Omri S., Behar-Cohen F., de Kozak Y. (2011). Microglia/macrophages migrate through retinal epithelium barrier by a transcellular route in diabetic retinopathy: role of PKC*ζ* in the Goto Kakizaki rat model. *The American Journal of Pathology*.

[41] Chatziralli I. P., Sergentanis T. N., Sivaprasad S. (2016). Hyperreflective foci as an independent visual outcome predictor in macular edema due to retinal vascular diseases treated with intravitreal dexamethasone or ranibizumab. *Retina*.

[42] Turkmen K., Ozcicek F., Ozcicek A., Akbas E. M., Erdur F. M., Tonbul H. Z. (2014). The relationship between neutrophil-to-lymphocyte ratio and vascular calcification in end-stage renal disease patients. *Hemodialysis International*.

[43] Ahsen A., Ulu M. S., Yuksel S. (2013). As a new inflammatory marker for familial Mediterranean fever: neutrophil-to-lymphocyte ratio. *Inflammation*.

[44] Dursun A., Ozturk S., Yucel H. (2015). Association of neutrophil/lymphocyte ratio and retinal vein occlusion. *European Journal of Ophthalmology*.

[45] Ilhan N., Daglioglu M. C., Ilhan O. (2015). Assessment of neutrophil/lymphocyte ratio in patients with age-related macular degeneration. *Ocular Immunology and Inflammation*.

[46] Kurtul B. E., Ozer P. A. (2016). The relationship between neutrophil-to-lymphocyte ratio and age-related macular degeneration. *Korean Journal of Ophthalmology*.

[47] Ulu S. M., Dogan M., Ahsen A. (2013). Neutrophil-to-lymphocyte ratio as a quick and reliable predictive marker to diagnose the severity of diabetic retinopathy. *Diabetes Technology & Therapeutics*.

[48] Bolayir A., Gokce S. F., Cigdem B. (2018). Monocyte/high-density lipoprotein ratio predicts the mortality in ischemic stroke patients. *Neurologia i Neurochirurgia Polska*.

[49] Aydin E., Ates I., Fettah Arikan M., Yilmaz N., Dede F. (2017). The ratio of monocyte frequency to HDL cholesterol level as a predictor of asymptomatic organ damage in patients with primary hypertension. *Hypertension Research*.

[50] Ancuta P., Wang J., Gabuzda D. (2006). CD16+monocytes produce IL-6, CCL2, and matrix metalloproteinase-9 upon interaction with CX3CL1-expressing endothelial cells. *Journal of Leukocyte Biology*.

[51] Murphy A. J., Woollard K. J. (2010). High-density lipoprotein: a potent inhibitor of inflammation. *Clinical and Experimental Pharmacology & Physiology*.

[52] Şatırtav G., Mirza E., Oltulu R., Mirza G. D., Kerimoğlu H. (2020). Assessment of monocyte/HDL ratio in branch retinal vein occlusion. *Ocular Immunology and Inflammation*.

[53] Villanueva D. L. E., Tiongson M. D., Ramos J. D., Llanes E. J. (2020). Monocyte to high-density lipoprotein ratio (MHR) as a predictor of mortality and major adverse cardiovascular events (MACE) among ST elevation myocardial infarction (STEMI) patients undergoing primary percutaneous coronary intervention: a meta-analysis. *Lipids in Health and Disease*.

[54] Ceyhun G., Engin M. Ç. (2021). The monocyte/high density lipoprotein cholesterol ratio (MHR) as an indicator of the need for amputation in patients with peripheral artery disease developing critical limb ischemia. *Angiology*.

